# Development of an Economical Method to Reduce the Extractable Latex Protein Levels in Finished Dipped Rubber Products

**DOI:** 10.1155/2017/9573021

**Published:** 2017-06-19

**Authors:** Ambegoda Liyanage Harini Amalka Perera, Bulathsinhalage Gayani Kanchana Perera

**Affiliations:** Department of Chemistry, Faculty of Science, University of Colombo, 03 Colombo, Sri Lanka

## Abstract

Natural rubber latex (NRL) allergy is caused by the extractable latex proteins in dipped rubber products. It is a major concern for the consumers who are sensitive to the allergenic extractable proteins (EP) in products such as NRL gloves. Objective of this research was to develop an economical method to reduce the EP in finished dipped NRL products. In order to reduce the EP levels, two natural proteases, bromelain from pineapple and papain from papaya, were extracted and partially purified using (NH_4_)_2_SO_4_. According to the newly developed method, different glove samples were treated with a 5% solution of each partially purified enzyme, for 2 hours at 60°C. Residual amounts of in treated samples were quantified using the modified Lowry assay (ASTM D5712-10). Bromelain displayed a 54 (±11)% reduction of the EP from the dipped rubber products, whereas it was 58 (±8)% with papain. These results clearly indicate that the selected natural proteases, bromelain, and papain contribute significantly towards the reduction of the total EP in finished NRL products. Application of bromelain enzyme for the aforementioned purpose has not been reported up to date, whereas papain has been used to treat raw NRL towards reducing the EP.

## 1. Introduction

Rubber* (Hevea brasiliensis)* is a tree of economic value mainly due to the milky latex which is the primary source of natural rubber [1]. Natural rubber latex (NRL) occurs in the latex vessels of the bark outside the phloem and the milky latex is collected by tapping the rubber tree bark [2]. NRL is a polymer of* cis*-1,4-isoprene. It is a milky, colloidal fluid is comprised of 30–40% of rubber hydrocarbon particles suspended in a serum together with 55–65% of water, 5% of other nonrubber substances such as 1.1–2% of proteins, lipids, resins, and sugars, and some metals (http://www.srilankabusiness.com/rubber/natural-rubber.html (accessed on April 10, 2015)).

The level of proteins in NRL will vary to some extent and its bulk is in the aqueous phase. Some EPs present in NRL have the potential to provoke an allergic reaction in some individuals. Over 200 different polypeptides have been identified in fresh NRL and 25% of them have been found to be responsible for allergic reactions caused by NRL [3].

NRL collected from rubber trees is processed via separate routes to make two different raw materials: latex concentrate and dry rubber which give rise to dipped rubber products and dry rubber products, respectively [4]. NRL gloves are the main type of dipped rubber product in the global market. The centrifugation, prevulcanization leaching, and postvulcanization leaching steps in the manufacturing process of NRL gloves remove a significant amount of the extractable proteins in NRL leading to reduced risk of protein allergies [5]. Yet, depending on the processing conditions used during the manufacturing process, different dipped rubber products have varying amounts of extractable proteins, even if those products are made from the same latex concentrate [6]. Some of these remaining residual extractable proteins are responsible for the allergic reactions observed among some of the dipped rubber product consumers [4].

NRL products are globally used among glove wearing healthcare workers, housekeeping personnel, and also children of every age group who may consume it in the forms of teats, baby soothers, and balloons. The main objective of wearing gloves is to get protection from infectious materials and harmful chemicals. It is widely acknowledged that NRL gloves are unsurpassed by any of the synthetic gloves because these contain excellent barrier protective capability and other superior physical properties [7]. Nonlatex synthetic gloves lack comfortable fit and have lower strength and endurance compared to NRL gloves [7]. Even though there are synthetic gloves in the market, the surgical gloves made from nonlatex materials are in low demand due to its high cost and low viability in large-scale daily use and mainly because the alternative materials used in synthetic gloves do not provide greater sensitivity and barrier against harmful irritants as the NRL gloves do [7]. However, the latex protein allergy associated with NRL gloves and other dipped rubber products could bring upon a negative impact on the dipped rubber product industry. It paves the way for diverting consumer attraction towards other substitutes such as synthetic gloves, silicone based teats, and baby soothers instead of latex based products which would eliminate their chances of facing a NRL allergic reaction.

With dipped latex products, the usual route of exposure to extractable latex proteins is through skin contact, especially in the presence of moisture [3]. However, if the product is powdered with cornstarch, a common powdering material, used for surgical gloves, it has been shown that the latex proteins can be adsorbed onto starch, become airborne, and offer an additional exposure route to the proteins through inhalation of the powder [8]. For this reason, powder-free gloves are preferred for the use in critical areas, such as hospitals where patients' immune response may be compromised. Upon exposure to these proteins, the body's immune system wrongly identifies these proteins as hostile invaders and activates the defense mechanisms. These defenses include the production of histamines, which in turn triggers an inflammatory response in the body. The consequences of this set of responses can range from a mild allergic reaction to a potentially fatal anaphylactic shock [9]. There are two main types of allergic reactions affecting certain individuals due to the use of NRL products. Latex protein hypersensitivity (type I) is caused by the residual water soluble latex proteins and chemical hypersensitivity (type IV) is caused by residues of chemicals used for processing of gloves [10]. It had been proven that extractable protein levels below 100 *µ*g g^−1^ in NRL products contain very low allergenic potential and this can be taken as the safe extractable protein level for NRL products [3]. Although nonlatex synthetic gloves available in the market do not contain any allergenic proteins, they do contain the same amount of curative and protective chemicals as NRL products, which can cause skin irritations and type IV chemical hypersensitivity [3]. Therefore, reduction of the extractable protein allergens from the NRL gloves and other dipped rubber products could make those suitable for any type of person. As NRL products are widely used around the world, it is important to pay immediate attention to reduce these allergens from NRL products before they enter into the global market.

Many efforts had been made by worldwide dipped rubber product manufacturers to reduce the extractable protein levels of their products. Nowadays numerous practices are incorporated in their manufacturing processes, such as adoption of improved leaching protocols during processing, use of low-protein lattices, chlorination, and polymer coating by incorporating a silica dispersion (http://www.kossan.com.my/products/gloves/surgical-procedures.html#top-page (accessed on June 9, 2017)). Some manufacturers use polyphenolic compounds such as tannins, which complex with proteins and precipitate them, and they could be washed away with a dilute solution of tannic acid to further remove the extractable proteins (Rubber Asia: The Complete Magazine on Rubber http://rubberasia.com/ (accessed July 27, 2015)). Another approach was carried out using eggshells from chickens, ducks, and birds. Pyrolysis of eggshells was done at 900°C for 2 hours transforming the calcium carbonate into calcium oxide. Then it was reacted with hydrochloric acid to form calcium chloride. Addition of calcium chloride and sodium dodecyl sulphate (SDS) into concentrated NRL has reported to effectively remove extractable proteins from NRL films [6].

It had been found that the extractable protein content of NRL gloves can also be reduced by washing the specimen with a solution containing a soluble silicate. Further improvement in protein reduction had been observed when an alkaline protease derived from* Bacillus* or* Aspergillus*, particularly a subtilisin, was mixed in the solution and it had resulted in further removing and making the remaining proteins less antigenic (http://www.google.com/patents/WO1997001581A1 (accessed on July 28,2015)).

A group of scientists at University of Maryland School of Medicine in Baltimore have tried to reduce the extractable antigenic protein content of commercial-grade NRL and finished products by treating the NRL with commercially available proteases produced from the fermentation of selected nonpathogenic strains of bacteria. The proteases that have been used were not revealed in the available sources. However, they have been able to successfully prove that the enzyme treatment of NRL alters the antigenic proteins associated with NRL by cutting them into smaller pieces and rendering them less immunogenic [2]. Similarly, researchers have tried to decompose the proteins in skim rubber by treating skim latex with liquid papain from papaya plant* (Carica papaya)* to enhance technological properties and aging characteristics of skim rubber [11].

Several studies have been done with papain to obtain high-quality low-protein rubber from field latex ((SJP-Repository: Enhancement of Deproteinization of* Hevea* Rubber by Maturation of Papain Treated* Hevea* Latex; (http://dr.lib.sjp.ac.lk/handle/123456789/857 (accessed on August 1, 2015)))). However, treatment of NRL and using it for the production of NRL products have not been a significantly successful approach towards producing finished dipped rubber products with low amounts of extractable proteins.

Therefore, development of a method to treat the finished dipped rubber products to achieve this goal seemed more attractive and effective. This possibility was investigated during this research by treating finished dipped rubber products with papain and bromelain proteases towards reducing their extractable latex proteins. Use of bromelain towards reduction of extractable proteins from NRL or NRL based products has not been reported up to date. NRL gloves were used as the main type of dipped rubber product during this research.

A few of the aforementioned methods involved the use of proteases because of their capability of hydrolyzing proteins. Natural enzymes such as papain and bromelain, which are nonspecific proteases, can digest longer peptides to smaller fragments, making them easy to be removed by few washing steps. During this research, efforts were made to develop a method to reduce the extractable latex proteins from finished dipped rubber products using papain and bromelain. This concept was investigated by treating NRL glove products with the two natural proteases, to test the possibility of incorporating a similar step in the dipped product manufacturing process at the industrial scale. Papaya* (Carica papaya)* and pineapple* (Ananas comosus)* are two rich natural sources of papain and bromelain, respectively [12, 13]. These two plants are found in abundance in many tropical countries and could be an economical option to obtain the aforementioned enzymes even for an industrial scale treatment process. Therefore, the newly developed treatment method involving these commonly found papain and bromelain proteolytic enzymes could be an effective and cost-effective method to bring out high-quality glove and other finished dipped rubber products with minimum levels of extractable proteins.

## 2. Materials and Methods

### 2.1. Extraction and Purification of Bromelain from* Ananas comosus* (Pineapple)

The crude pineapple extracts were obtained from the pineapple stem waste. The plant parts were processed using a kitchen blender and the mixture was filtered through a piece of cheesecloth. The resultant solution was centrifuged at 2000 r.p.m. for 20 minutes at 4°C to remove any insoluble material [14]. Solid (NH_4_)_2_SO_4_ was added to the cooled crude pineapple extract maintained at 0°C until the (NH_4_)_2_SO_4_ percentage was 40%. After adding the desired amount of (NH_4_)_2_SO_4_, the extract was centrifuged at 2000 r.p.m. for 20 minutes at 4°C. The resulting pellet was resolubilized in distilled water. Partially purified stem bromelain was stored at 4°C until further use [15]. Percentage yield was calculated and the concentration of partially purified bromelain enzyme was found using the Bradford assay.

### 2.2. Extraction and Purification of Papain from* Carica papaya *(Papaya)

Fully green unripe papaya fruits were collected in the early morning. Surface of the papaya fruits were cleaned using 70% ethanol and several longitudinal incisions which were not deeper than 2 mm were made using a stainless steel sterilized razor blade. Papaya latex was collected into a sterilized Petri dish to minimize any microbial contaminations. Collected latex was stored at −20°C until further purification was done [16]. Thawed papaya latex was mixed with 40 mM cysteine at a ratio of 3 : 1 (w/v). The suspension was adjusted to pH 5.6 using 6 M HCl and then was stirred for 15 minutes at 4°C. The mixture was filtered and the pH of the filtrate was adjusted to 9.0 using 6 M NaOH. The insoluble material was removed by centrifugation at 3000 r.p.m. for 30 minutes at 4°C [16]. The supernatant was separated and protein precipitation was carried out with a 45% (NH_4_)_2_SO_4_ solution. The salt-enriched solution was slowly stirred at 4°C for 30 minutes. The precipitate was collected by centrifugation and it was redissolved using distilled water. Partially purified papain enzyme was stored at 4°C until further use [16]. Percentage yield was calculated and the concentration of partially purified papain enzyme was found using the Bradford assay.

### 2.3. Bradford Assay

A volume of 800 *µ*L of each BSA standard or the test sample solution was pipetted into a dry test tube. A volume of 200 *µ*L of the Bradford Reagent was added to each tube and was mixed thoroughly. The samples were incubated at room temperature for 10 minutes. The absorbance of the samples at 595 nm was measured using a UV visible spectrophotometer (http://www.bio-protocol.org/e45 (accessed on May 14, 2015)). The unknown concentrations were found using the standard curve developed using the BSA protein standards.

### 2.4. Confirmation of the Protease Activity of Partially Purified Papain and Bromelain Enzymes through the Egg White Digestion Test

A piece of boiled egg white, with a known weight (~1 g), was treated with 2 mL of 5% papain, 5% bromelain, or distilled water control. The mixtures were incubated for 2 hours at 60°C. After 2 hours, the mixtures were filtered and the surface water on the egg white pieces were removed using blotting paper without applying force. The remaining weight of the egg white pieces was measured for each sample and the percentage weight reduction for each sample was calculated [17]. All experiments were carried out in triplicate.

### 2.5. Treatment of Glove Samples with Protease Enzymes

Glove samples, cut into small pieces, weighing 2 g each, were treated with 10 mL of a 5% (w/v) solution of the partially purified protease (bromelain or papain). Distilled water was used as the control solution. Glove samples were incubated with the enzyme solution for 2 hours at 60°C. Then the glove samples were subjected to 4 × 15 minutes distilled water washes (a volume of 50 mL per wash) carried out at room temperature. Residual extractable proteins of the enzyme treated glove samples were extracted and quantified according to the modified Lowry assay described in ASTM D5712-10 (Standard Test Method for Analysis of Aqueous Extractable Protein in Natural Rubber and Its Products Using the Modified Lowry Method). All experiments were carried out in triplicate and respective ±SEM (Standard Error of Mean) is calculated for each and presented along with the average value in Results and Discussion and in detail in Tables 4 and 5. The method of extraction and quantification of extractable proteins from treated glove samples according to the ASTM D5712-10 is indicated below.

Another set of glove treatment experiments was carried out using the crude bromelain and papain enzymes instead of the partially purified enzymes to investigate the importance of enzyme purification step.

### 2.6. Extraction of Latex Proteins from Dipped Rubber Products

Each treated glove sample was placed in a polypropylene vessel with a 10 mL portion of 1x PBS extraction buffer making sure that all the surfaces of the test specimen were exposed to the extraction solution. The extraction was carried out at 25°C for 2 hours in a shaker set to a speed of 200 r.p.m. The test specimen was removed from the extraction solution and the extract was transferred into a polypropylene centrifuge tube and was centrifuged for 15 minutes at 3000 r.p.m. to remove the particulate matter. The supernatant liquid was collected and the protein quantification was carried out within 24 hours. This latex protein extract was stored at 4°C until being used [18].

### 2.7. Acid Precipitation of Latex Proteins

A volume of 1 mL of each latex protein extract was accurately transferred into a 1.5 mL polypropylene tube and 0.1 mL of DOC was added. The resulting solution was mixed and was allowed to stand for 10 minutes. A volume of 0.2 mL of a freshly prepared 50 : 50 TCA : PTA solution was added to acid precipitate the proteins. The solution was well mixed and was allowed to stand for an additional 30 minutes before centrifugation. Then, the acid precipitate was centrifuged at 3000 r.p.m. for 20 minutes. The supernatant liquid was decanted. The protein pellet was stored at 4°C and was analyzed within 24 hours [18].

### 2.8. Resolubilization of Latex Protein Precipitates

A volume of 0.25 mL of 0.2 M NaOH solution was added to each tube containing the pelleted proteins to redissolve the precipitated proteins. If necessary, the mixture was vortexed or another known quantity of 0.2 M NaOH solution was added up to a total of 1 mL to ensure that the precipitated protein was completely redissolved to give a clear solution [18].

### 2.9. Modified Lowry Assay for Quantification of Extracted Proteins

A volume of 300 *µ*L of the redissolved latex protein solution, a BSA protein standard, or the reagent blank (0.2 M NaOH) was added to a volume of 625 *µ*L of alkaline copper tartrate. The resulting solution was well mixed and allowed to stand for 15 minutes at room temperature. A volume of 75 *µ*L of 1x Folin-Ciocalteu Phenol reagent was added to the mixture and it was thoroughly mixed immediately. The solution was allowed to stand for 30 minutes at room temperature. The absorbance of the resulting solution was measured at 750 nm using a UV visible spectrophotometer and the unknown protein concentrations were found using a BSA standard curve [17, 18].

In addition to the NRL gloves, an investigation of the extractable latex protein contents on few other selected dipped rubber products was also carried out using the above extraction protocol and modified Lowry assay.

## 3. Results and Discussion

Observations of each aforementioned experiment were recorded and they were further analyzed as follows.

### 3.1. Investigation of the Extractable Protein Levels in a Selected Set of Dipped Rubber Products in the Market

**Figure 1:**
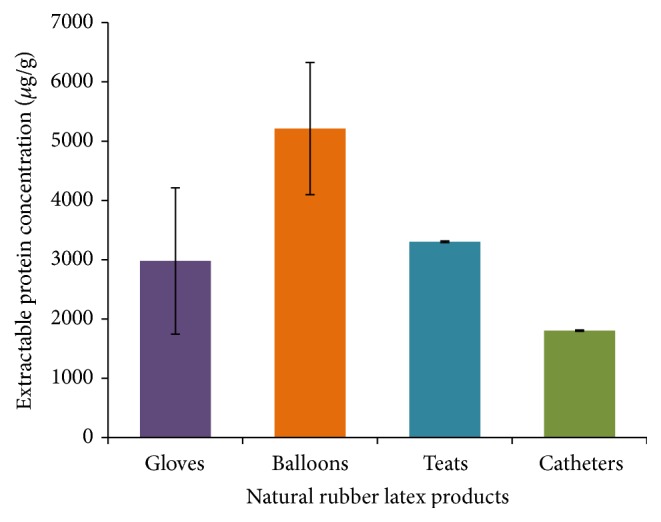
The average extractable protein levels in different NRL dipped products available in the market.

In order to obtain an idea about the levels of extractable latex proteins in selected dipped rubber products in the market, a set of different latex based products were analyzed and the results of this investigation are shown in Table 1 and Figure 1. All the glove samples and balloon samples that were analyzed during this study were made of 100% NRL and these products were quite abundant in the market. Even though a 100% NRL based teat samples were analyzed during this work, those are not commonly available in the market currently. Majority of the teats available consist of a mixture of NRL and silicone. The catheter samples used in this study were also made out from a mixture of NRL and silicone. All samples were analyzed in triplicate and those results are shown in Table 1.

According to these results, the balloons have the highest amount of extractable proteins and then the teats and gloves in the descending order. Lowest amount of extractable proteins was recorded for the catheter sample which was made of a mixture of NRL and silicone. Above data shows that the dipped rubber products currently available in the local market contain a significant amount of latex proteins which may provoke allergic conditions in consumers. Furthermore, the amounts of extractable proteins in the dipped rubber products seemed to vary vastly depending on the product type and the manufacturer. Therefore, it is important to address this issue and develop a new consistent method to reduce the amounts of residual extractable latex proteins in finished dipped rubber goods to improve their quality.

### 3.2. Purification and Testing of Protease Enzymes

The percentage yield for the partially purified stem bromelain enzyme was around 2% and its concentration was quantified to be 3997 (±604) according to the Bradford assay.

It was observed that the mornings (between 6 a.m. and 9 a.m.) were better to collect a considerable amount of papaya latex than later during the day. Upon partial purification of crude papaya latex using 45% (NH_4_)_2_SO_4_ solution, the papain yield was about 5% and its concentration was around 3950 (±17) as quantified by the Bradford assay.

Prior to being used for the glove treatment experiments, both bromelain and papain protease activities were confirmed using an egg white digestion test. Please refer to Table 2.

In the egg white digestion test, albumin which is the major protein in the egg white, is digested by the protease enzyme of interest. As albumin is hydrolyzed a weight reduction was observed and this was correlated with the activity of the protease enzyme of interest. Bromelain resulted in a 15 (±1)% weight reduction in the egg white digestion test whereas, for papain, it was 26 (±2)%.

### 3.3. Glove Treatment Experiments Carried out with Protease Enzymes

Glove treatment experiments were carried out with 5% solutions of crude and partially purified bromelain and papain solutions. The percentage reduction of the extractable proteins from the glove samples treated for 2 hours at 60°C with the respective enzymes is indicated in Table 3. After the enzyme treatment step, the hydrolyzed smaller proteins and peptides were washed away from the latex product via four 15-minute washes carried out with distilled water at room temperature.

According to the results in Table 3, glove indicated a significant reduction of extractable proteins upon treatment with partially purified papain and bromelain enzymes. Partially purified papain resulted in a 58% reduction of the extractable proteins from the treated glove samples whereas this was 54% for the bromelain treated glove samples. However, the removal of extractable proteins from the glove samples treated with crude proteases was much less significant and it was 12% and 14% reduction for papain and bromelain enzymes, respectively. This could be correlated with the enzyme activity observed during the egg white digestion tests carried out with these enzymes (Table 2). The partially purified enzymes clearly indicated a significantly higher protease activity compared to the crude enzyme extracts. Therefore, it can be suggested that the partial purification of bromelain and papain using (NH_4_)_2_SO_4_ could contribute towards better removal of extractable proteins from dipped rubber products during the enzyme treatment step. Please refer to the appendix for data tables (Tables 4 and 5) representing all trials for above experiments summarized in Table 3 along with respective ±SEM (Standard Error of Mean).

**Figure 2:**
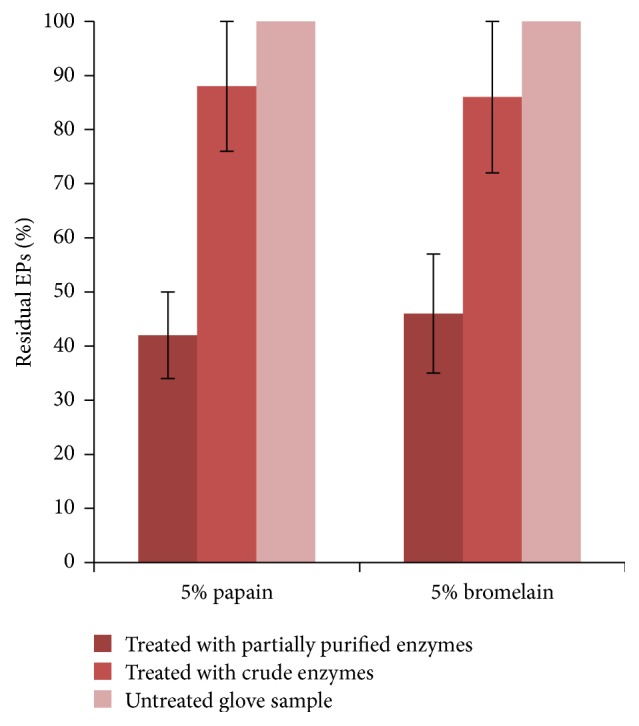
Residual extractable proteins of glove samples treated with partially purified and commercial protease enzymes respectively.

According to the summarized data presented in Figure 2, it is clearly evident that both partially purified bromelain and papain enzymes contribute successfully towards the removal of extractable proteins from the glove samples. Even though the partially purified papain resulted in a slightly higher reduction of the extractable proteins from the treated glove samples compared to the reduction resulted by partially purified bromelain sample, bromelain could be a more economical option for the specified purpose as it is completely isolated from the stem waste products generated during pineapple consumption. However, if papain is to be used, since the young fruits need to be injured while collecting the necessary latex, that could be practically disadvantageous compared to the situation with extracting stem bromelain. Therefore, bromelain can be identified as an inexpensive and more appropriate resource for this application.

## 4. Conclusion

Natural rubber latex (NRL) allergy is a critical health issue faced by hypersensitive, dipped rubber product consumers around the world. During the investigation of extractable protein levels in a set of commercially available dipped rubber products in the local market, it was found that they contain varying amounts of extractable latex proteins at quantities much greater than the recommended safety levels.

Treatment of NRL gloves with 5% solutions of partially purified bromelain or papain enzymes for 2 hours at 60°C resulted in 54 (±11)% and 58 (±8)% reduction of the extractable latex proteins from the treated glove samples. Bromelain was partially purified from pineapple stem waste using 40% (NH_4_)_2_SO_4_ and partially purified papain was obtained from papaya fruit latex using 45% (NH_4_)_2_SO_4_. The gloves treated with crude bromelain and papain enzymes only resulted in 14 (±4)% and 12 (±1)% reduction of the extractable latex proteins from the treated glove samples, respectively. Therefore, it can be recommended to treat finished dipped rubber products with partially purified bromelain or papain solutions for 2 hours at 60°C to reduce their residual extractable protein levels in an efficient and economical manner. The finding of this research will contribute towards producing dipped rubber products with low allergenic potential and improved quality, while keeping the production cost at a low level.

## Figures and Tables

**Table 1:** Average extractable latex proteins of different NRL dipped products in the market.

NRL product	Concentration of extractable proteins (*µ*g g^−1^)
Gloves	2979 (±1233)
Balloons	5212 (±1114)
Teats	3303 (±14)
Catheters	1803 (±11)

**Table 2:** Egg white digestion test results showing the activity of extracted bromelain and papain (*n* = 4).

Enzyme treated with	Egg white weight reduction as a %
5% partially purified papain	26 (±2)
5% partially purified bromelain	15 (±1)
Crude papain	14%
Crude bromelain	10%

**Table 3:** Results of the glove treatment experiments carried out with bromelain and papain enzymes.

Condition	EP after treatment (*µ*g g^−1^)			
	Crude bromelain	Partially purified bromelain	Crude papain	Partially purified papain
Glove sample treated with enzyme	2770 (±1036)	576 (±333)	2827 (±925)	512 (±459)
Glove sample treated with distilled water (control)	3178 (±973)	1079 (±647)	3178 (±973)	1079 (±647)
Percentage reduction of extractable proteins (EP)	14 (±4)	54 (±11)	12 (±1)	58 (±8)

**Table 4:** Results of the glove treatment experiments carried out with partially purified papain and bromelain enzymes.

Sample	Concentration of extractable proteins			
	Trial 1 (*µ*g g^−1^)	Trial 2 (*µ*g g^−1^)	Trial 3 (*µ*g g^−1^)	Average (±SEM)
Untreated glove sample	655	480	1620	
Glove sample treated with partially purified papain	312	186	1037	
Glove sample treated with partially purified bromelain	282	205	1240	
Glove sample treated with distilled water (control)	788	628	1820	

Percentage reduction of extractable proteins (EP)			
Papain treated (%)	60	70	43	58 (±8)
Bromelain treated (%)	64	67	32	54 (±11)

**Table 5:** Results of the glove treatment experiments carried out with crude papain and bromelain enzymes.

Sample	Concentration of extractable proteins			
	Trial 1 (*µ*g g^−1^)	Trial 2 (*µ*g g^−1^)	Trial 3 (*µ*g g^−1^)	Average (±SEM)
Untreated glove sample	3360	4255	2220	
Glove sample treated with crude papain	2840	3745	1895	
Glove sample treated with crude bromelain	2640	3865	1805	
Glove sample treated with distilled water (control)	3200	4140	2195	

Percentage reduction of extractable proteins (EP)				
Papain treated (%)	11	10	14	12 (±1)
Bromelain treated (%)	18	7	18	14 (±4)

## Abbreviations

ASTM: American standard test method
BSA: Bovine serum albumin
DOC: Deoxycholate
EP: Extractable protein
NRL: Natural rubber latex
PBS: Phosphate buffered saline
PTA: Phosphotungstic acid
r.p.m.: Rounds per minute
RT: Room temperature
SEM: Standard Error of Mean
SL: Sri Lanka
TCA: Trichloroacetic acid.
